# Parasite threshold associated with clinical malaria in areas of different transmission intensities in north eastern Tanzania

**DOI:** 10.1186/1471-2288-9-75

**Published:** 2009-11-12

**Authors:** Bruno P Mmbando, John P Lusingu, Lasse S Vestergaard, Martha M Lemnge, Thor G Theander, Thomas H Scheike

**Affiliations:** 1 Department of Biostatistics, University of Copenhagen, Copenhagen, Denmark; 2 National Institute for Medical Research, Tanga Centre, Tanga, Tanzania; 3 Centre for Medical Parasitology, Institute for Medical Microbiology and Immunology, University of Copenhagen, Copenhagen, Denmark

## Abstract

**Background:**

In Sub-Sahara Africa, malaria due to *Plasmodium falciparum *is the main cause of ill health. Evaluation of malaria interventions, such as drugs and vaccines depends on clinical definition of the disease, which is still a challenge due to lack of distinct malaria specific clinical features. Parasite threshold is used in definition of clinical malaria in evaluation of interventions. This however, is likely to be influenced by other factors such as transmission intensity as well as individual level of immunity against malaria.

**Methods:**

This paper describes step function and dose response model with threshold parameter as a tool for estimation of parasite threshold for onset of malaria fever in highlands (low transmission) and lowlands (high transmission intensity) strata. These models were fitted using logistic regression stratified by strata and age groups (0-1, 2-3, 4-5, 6-9, and 10-19 years). Dose response model was further extended to fit all age groups combined in each stratum. Sub-sampling bootstrap was used to compute confidence intervals. Cross-sectional and passive case detection data from Korogwe district, north eastern Tanzania were used.

**Results:**

Dose response model was better in the estimation of parasite thresholds. Parasite thresholds (scale = log parasite/*μ*L) were high in lowlands than in highlands. In the lowlands, children in age group 4-5 years had the highest parasite threshold (8.73) while individuals aged 10-19 years had the lowest (6.81). In the highlands, children aged 0-1 years had the highest threshold (7.12) and those aged 10-19 years had the lowest (4.62). Regression analysis with all ages combined showed similar pattern of thresholds in both strata, whereby, in the lowlands the threshold was highest in age group 2-5 years and lowest in older individuals, while in the highlands was highest in age group 0-1 and decreased with increased age. The sensitivity of parasite threshold by age group ranged from 64%-74% in the lowlands and 67%-97% in the highlands; while specificity ranged between 67%-90% in the lowlands and 37%-73% in the highlands.

**Conclusion:**

Dose response model with threshold parameter can be used to estimate parasite threshold associated with malaria fever onset. Parasite threshold were lower in older individuals and in low malaria transmission area.

## Background

Malaria is a major cause of ill health in Africa, especially south of the Sahara, where it takes its greatest toll in young children and pregnant women. About 90% of all malaria deaths in the world today occur in sub-Saharan Africa, where the most virulent species of the parasite, *Plasmodium falciparum *flourishes. According to health facility statistics, in Tanzania malaria is the leading cause of morbidity and mortality and accounts for about 30% and 15% of hospital admissions and deaths, respectively [1]. The major cause of malaria related deaths is severe anaemia [2] and complicated malaria, which can be attributed to delay in seeking health care [3], inadequate functional referral system, poor quality of health care and emergence of resistance to commonly used antimalarial drugs [4].

Control of malaria currently relies on chemotherapy [5] and mosquito controls [6,7]. Other control strategies would be vaccination which could target different stage of parasite development. Several vaccines are at different phase of clinical development [8], however, none is yet licensed.

Malaria controls face lot of challenges due to parasite and host dynamics, which includes resistance to both insecticides and antimalarial drugs, and slow development of immunity [5]. The acquired malaria immunity varies with the level of exposure to parasites; hence individuals living in holo-endemic areas tend to acquire immunity sooner than those in less endemic areas. Thus, individuals of similar age living under different malaria transmission intensity can vary considerably with respect to their vulnerability to infection [9].

Evaluation of efficacy of different interventions requires precise definition of endpoints, but the problem is that clinical features of malaria are not specific, for example, presence of parasite alone or parasite with fever are not adequate definition of clinical malaria [10,11]. However, high parasite density is more likely to coincide with fever [10,11], so setting criteria for definition of clinical malaria should be done with care as it may lead to some cases being misclassified; for both false positives and negatives resulting in decreased specificity and sensitivity. Low sensitivity will result if true positive cases are misclassified as negatives which could arise due to measurement errors or case definition being too stringent.

The predominant variables in definition of malaria disease are parasite density and measured fever. For example Smith et al. [10] used logistic model to model the fever attributable to different levels of parasite densities in endemic areas. They further used sensitivity-specificity analysis to estimate the parasite threshold associated with fever. Rogier et al. [12], modeled the parasite threshold as a step function to relate to the risk of fever. Other models that could be used include dose response models which has been used in toxicology studies [13-15]. These models consider different level of doses, to establish a dose below which no effect occur to organs or system, or in extreme the death of an organism. The theory of threshold in toxicology is the minimum dose that its effect is likely to be similar to the background (i.e similar to dose zero) [15].

Main aim of this paper is to determine *Plasmodium falciparum *parasite threshold associated with fever in high and low malaria transmission intensity areas. In the study area malaria transmission intensity is influenced by altitude, where the lowlands are characterized by a high transmission and highlands by low transmission [16]. The motivation behind is that, an individual develops malaria fever when a parasite density exceeds certain threshold, and this is assumed to vary with transmission intensity and age of an individual.

## Methods

### Data description

This involved a case-control study in individuals aged below 20 years in six villages (Kwamasimba, Kwamhanya, Magundi, Kwashemshi, Mng'aza and Mkokola) of Korogwe District, in Tanzania. These villages were under passive case detection (PCD) of malaria fevers as detailed elsewhere [17,18]. The first three villages are located in the highland and last three in lowland areas. In the PCD system, community health workers were trained to manage uncomplicated malaria episodes on clinical grounds, whereby for individuals who presented with history of fever in the past two days or with axillary temperature ≥ 37.5°*C*, a morbidity questionnaire was completed. Blood smear for malaria parasite detection was taken and first line antimalarial drug was administered. Pregnant women and individuals with signs of severe malaria or other features suggesting other complications were referred to nearby health facility or Korogwe district hospital [17]. Sulphadoxine pyrimethamine was used as the first line up to January 2007, while Artemether/lumefantrine was introduced in the villages in February 2007 following the change of antimalarial drug policy in Tanzania.

Cross-sectional malariometric surveys were conducted in these villages during low malaria transmission (Sep/Dec) and high transmission (March/June) seasons, where assessment of clinical features were done for all consenting individuals, and then blood samples for malaria parasite detection by thick and thin blood smears were taken.

Cases were detected from individuals under PCD and controls were individuals recruited during cross-sectional malaria surveys [19]. Individuals who reported with measured fever (axillary temperature ≥ 37.5°*C*) to the community health workers with intention to be treated for malaria in the six villages qualified as cases [20].

In the data set used, two villages (Kwamasimba from highland and Mkokola from lowland) started PCD in January 2003 while the rest were introduced in the system from January 2006 and all were followed to December 2007. A total of 7 cross-sectional surveys were done in villages where PCD started in 2003 and 4 in villages included in 2006. Malaria parasites were counted against 200 white blood cells (WBC), and a blood smear was declared negative after examination of 100 high power fields. Parasite counts/200 WBC were converted to parasites per microliter by multiplying by 40 assuming that 1 *μ*L of blood of normal person contains 8000 WBC. Parasite counts were normalized by natural log transformation.

**Figure 1:**
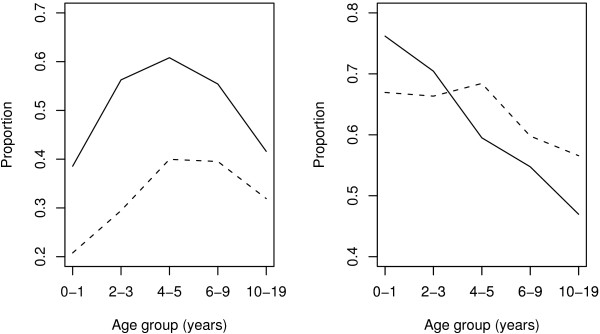
**Proportions of individuals with *Plasmodiumfalciparum *parasite (left) and fever among parasitaemic (right) by age group from PCD data**. Solid and dashed lines represent lowlands and highlands, respectively.

*Plasmodium falciparum *malaria parasite prevalence based on 6 cross sectional surveys conducted from 2005-2007 in the two strata, showed that in the lowlands the overall parasite prevalence was 37.4% while in the highlands it was 11.0%. Children below 5 years had a prevalence of 30.2% and 10.0%, 5-9 years had 46.9% and 11.8% while those in age group 10-19 years had 36.5% and 11.6% in lowlands and highlands, respectively. Distribution of *Plasmodium falciparum *parasite rate and fever according to age groups from the PCD data in the two strata, showed that children aged 4-5 years had the highest parasite rate, while among those infected with *Plasmodium falciparum*, individuals aged below 2 years had the highest rate of fever, see Figure 1.

The ethical clearance was granted by the Medical Research Coordinating Committee of the National Institute for Medical Research, Tanzania.

### Parasite threshold models formulation

#### Notation

Let *y*_*i*_, *i *= 1, 2,... *n *be a random variable which takes value of (*Y *= 1) if a fever episode is observed and (*Y *= 0) otherwise, with probability *Pr*(*Y*_*i *_= 1) = *γ*_*i *_and *Pr*(*Y*_*i *_= 0) = 1 - *γ*_*i*_. Let *p*_*i *_denote the log parasite density observed in symptomatic and asymptomatic individuals, where *n *is the number of observations. Let *τ *be a parasite threshold for an individual to develop malaria fever (clinical malaria).

#### Threshold models

The *n *response variables *y*_*i*_'s given parameter (*θ*) can be fitted as a binomial random variables by a joint distribution given by:

and log-likelihood function given by

Relation to the explanatory variable(s) is through a *logit *link function, such that

where *α *and *β *are parameter estimates and *p*_*i *_is log parasite density. However, our aim is the parasite threshold (*τ*) which is most likely to coincide with malaria fever. In modeling the parasite threshold, two approaches are considered: (1) use of a step function model, where individuals with parasite below a threshold (*τ*) are considered as healthy and otherwise as diseased group. The step function model takes the following form:(1)

where *p*_*i *_is the log parasite density, *τ *> 0 is the threshold, *I *is an indicator variable (0 if *p*_*i *_≤ *τ *and 1 if *p*_*i *_>*τ*), and *α *and *β *are location and scale parameters.

In (2), we consider malaria fever to be a function of continuous parasite density. In this case, fever episode are modeled as a continuous function of difference of parasite density and threshold parameter. Probability of fever in individuals with parasite less or equal to *τ *is assumed to be constant, *α *(which corresponds to background) and that above the *τ*, the probability increases with the increase in the difference of log parasite density and threshold parameter. The model is defined as:(2)

where *p*_*i*_, *τ *> 0, *α *and *β *are similar as above. In both cases the probability of clinical malaria in individuals with parasite below *τ *is logit^-1^(*α*), which corresponds to the background (i.e the probability of fever is considered to be similar between parasitaemic and non parasitaemic individuals). It should be noted that the assumption of abrupt change in reaction to infection following a certain level of parasite density is only used for modeling purposes but does not necessarily present what happens in a host.

#### Estimation of threshold by regression

To investigate if *τ *depend on age, a regression model can be fitted for different ages (age groups) combined, and then test if there is significant difference in the slope parameter. If we let the mean age of group *j *be *A*_*j*_, *j *= 1,..., *J*, and assume *τ *to show a similar trend as parasite rate shown in Figure 1, which was also shown elsewhere [12], and  be the mean age squared of group *j*, then *τ *can be modeled as a function of *A*_*j *_and  with parameters *θ *= (*θ*_0_, *θ*_1_, *θ*_2_). Otherwise, if *θ*_2 _is not significant then the effect of A* will be excluded from estimation of *τ *parameter. For simplicity, modeling will be done separately for each stratum as follows:

So, we can extend model 2 by inserting the estimated value of *τ *and get:

where *β*_1 _is the parameter measuring the excess risk of fever when age (or mean age) increase by one year, *β*_2 _is similar as *β *in model 2. If we let *Z*_*ij *_= (*p*_*ij *_- *τ*_*j*_), the model simplifies to(3)

#### Modeling fevers using non-threshold model

We used non linear model to fit the effect of parasite density on fever in each age group. The model was used for the purpose of determining how best the above threshold models (model 1 & 2) fits the data when compared to classical methods. Model considered is(4)

where *f*(*p*_*ij*_) = *β*(*p*_*ij*_)^*ν *^is a monotone increasing function of parasite density *p*_*ij*_. This model has been shown to fit well the relation of fever and parasite density better than the normal regression of fevers on parasites or log parasites, see [10]. We used Box-Tidwell [21] method to find the maximum values for *β *and *ν*. Performance of the models (1,2 and 4) were compared by using Akaike information criteria (AIC).

### Models fitting

The parameter of interest is *τ *which is unknown and cannot be estimated explicitly, we used profile likelihood method in fitting models 1 and 2. In profile likelihood, let *l*(*θ *: *p*_*i*_) = *l*(*τ, α, β *: *p*_*i*_) be a log likelihood function, then we can compute the maximum likelihood estimators for *α *and *β *which maximizes the log likelihood function for all value of *τ*, i.e

Maximum value of *τ *can then be obtained from a plot of  against *τ*.

Models 3 were fitted using simulated annealing procedure, optim with method option SANN from R statistical package. Confidence intervals for *τ *was obtained by subsampling bootstrap method [22], because models are not smooth in the threshold parameter. The convergence rate of the threshold parameter is *n*, that is *n*( - *τ*) converges in distribution [23].

### Simulation studies

We simulated 300 data sets, each with *n *= 2000 observations from a negative binomial distribution with mean, *μ *= 100 and scale parameter (0.03 and 0.1), to give a prevalence of 20% and 50% which mimics the distribution of parasites in a host [24] from lowlands and highlands, respectively. The simulated samples were multiplied by 40 and then log transformed to resemble unit used in quantification of malaria parasites (parasites/*μ*L) in log scale. We generated a binomial random variable from model (2). The binomial random variable was assigned a probability of occurrence to be *γ*_*i *_= logit^-1^(*α*) if *p*_*i *_≤ *τ *and *γ*_*i *_= logit^-1^(*α *+ *β*(*p*_*i *_- *τ*)) if *p*_*i *_>*τ*. We set different values of *τ *to be 4.4, 5.1, 6.0 and 9.0. The values of an *α *was set to be -1 and 0 while that of *β *was set to 0.5, 1 and 2. The choices of these parameters (*α*, *β*) were used to give different sampling probabilities of choosing cases and controls. To compare performance of the two models, we used root mean square error (RMSE) statistic. This statistic measures the differences between values predicted by a model estimator against the actual value. The RMSE of an estimator is defined as the square root of the mean squared error (MSE) of . If it is an unbiased estimator, then MSE is identical to the variance.

So, the mean square error of  is:

The MSE can be expressed in terms of variance and bias as

where  is the estimator and *τ *is actual parameter. The *MSE *is unknown and can be estimated by a sample mean as

where *τ*_*i *_is the realizations of the estimator  of size *n*. Model with minimum RMSE is chosen as the model with best fit.

### Sensitivity and Specificity of threshold parameter

We assessed the sensitivity and specificity of threshold in predicting malaria fever. We defined a positive test (*E*_1_) when parasite was above *τ*, otherwise a test was negative (*E*_0_); while individuals with fever were defined as a disease group *D*_1 _and those without fever as non-disease group *D*_0_. Case-control study can be used in estimation of sensitivity and specificity but not predictive values, since the latter require a prevalence of disease to be known or estimated from the data, and this cannot be obtained from our design [25]. So, for group *j *= 1,...,5 the sensitivity and specificity is given by:

where P is the probability.

## Results

**Figure 2:**
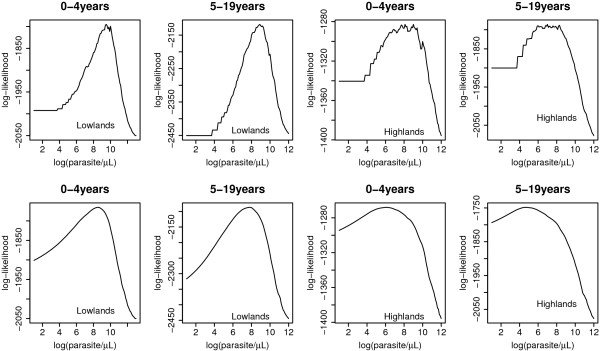
**Profile likelihood for log(parasite/*μ*L) in individuals aged 0-4 and 5-19 years in lowlands and highlands**. First rows is produced by model 1 and the second by model 2.

Results show that model (2) had higher log-likelihood in all estimates than model (1), see for example Figure 2, and simulation results (Table 1) which shows that model (2) had considerably lower bias. We also compared models (1) and (2) to that of model (4) to see how they best fit the data, and we found model (2) was doing well since in almost all age groups, it had the lowest AIC (Table 2). Estimated odds ratio from Table 2 cannot be compared because they explain different relation, example in the model 1, is the odds between those with log parasite density above the threshold and below the threshold, while in model 2 is the odds ratio of fever when log parasite density increases by 1 above the threshold.

**Table 1:** Parameter estimates from simulation of log transformed random variables from negative binomial distribution with size = (0.03 and 0.08) and mean = 100

			size = 0.03 and mean = 100						size = 0.1 and mean = 100					
			
Parameters			Model 1			Model 2			Model1			Model 2		
*α*	*β*	*τ*		RMSE	Bias		RMSE	Bias		RMSE	Bias		RMSE	Bias
0.00	0.50	4.40	6.28	2.00	1.88	4.39	0.54	0.01	4.77	0.38	0.37	4.39	0.35	0.01
0.00	0.50	5.10	6.88	1.93	1.78	5.07	0.58	0.03	5.91	0.82	0.81	5.08	0.40	0.02
0.00	0.50	6.00	7.81	1.95	1.81	6.10	0.64	0.10	6.85	0.88	0.85	5.99	0.45	0.01
0.00	0.50	9.00	9.94	1.13	0.94	9.05	0.71	0.05	9.79	0.95	0.79	9.05	0.59	0.05

0.00	1.00	4.40	5.77	1.46	1.37	4.44	0.35	0.04	4.79	0.40	0.39	4.41	0.23	0.01
0.00	1.00	5.10	6.42	1.40	1.31	5.09	0.38	0.01	5.91	0.82	0.81	5.10	0.23	0.00
0.00	1.00	6.00	7.25	1.33	1.25	6.00	0.38	0.00	6.88	0.90	0.88	5.99	0.23	0.01
0.00	1.00	9.00	9.84	0.96	0.84	9.00	0.43	0.00	9.68	0.75	0.68	9.04	0.30	0.04

0.00	2.00	4.40	5.18	0.87	0.78	4.45	0.26	0.05	4.79	0.39	0.39	4.42	0.14	0.01
0.00	2.00	5.10	5.93	0.88	0.83	5.12	0.28	0.02	5.82	0.74	0.72	5.10	0.15	0.00
0.00	2.00	6.00	6.79	0.84	0.78	6.03	0.28	0.03	6.72	0.75	0.72	5.99	0.16	0.01
0.00	2.00	9.00	9.64	0.70	0.64	9.02	0.33	0.02	9.52	0.56	0.52	9.00	0.20	0.00

-1.00	0.50	4.40	6.66	2.36	2.26	4.41	0.47	0.01	4.79	0.40	0.39	4.38	0.35	0.02
-1.00	0.50	5.10	7.29	2.29	2.19	5.08	0.49	0.02	5.94	0.85	0.84	5.09	0.36	0.01
-1.00	0.50	6.00	7.90	2.01	1.90	5.98	0.56	0.02	6.90	0.91	0.90	6.01	0.40	0.01
-1.00	0.50	9.00	10.00	1.19	1.00	9.06	0.74	0.06	9.81	1.01	0.81	9.01	0.60	0.01

-1.00	1.00	4.40	5.98	1.63	1.58	4.41	0.30	0.01	4.80	0.40	0.40	4.42	0.19	0.01
-1.00	1.00	5.10	6.66	1.62	1.56	5.11	0.32	0.01	5.96	0.86	0.86	5.10	0.20	0.00
-1.00	1.00	6.00	7.50	1.56	1.50	5.99	0.35	0.01	6.94	0.94	0.94	6.00	0.21	0.00
-1.00	1.00	9.00	10.00	1.08	1.00	9.01	0.44	0.01	9.77	0.84	0.77	8.96	0.35	0.04

-1.00	2.00	4.40	5.37	1.01	0.97	4.42	0.22	0.01	4.80	0.40	0.40	4.41	0.14	0.01
-1.00	2.00	5.10	6.07	1.01	0.97	5.11	0.24	0.01	5.92	0.83	0.82	5.11	0.14	0.01
-1.00	2.00	6.00	6.92	0.96	0.92	6.01	0.23	0.01	6.86	0.87	0.86	6.00	0.14	0.00
-1.00	2.00	9.00	9.72	0.77	0.72	8.98	0.25	0.02	9.60	0.63	0.59	9.01	0.15	0.01

**Table 2:** Estimates of parameters (*τ *= parasite threshold, *υ *= power, *β *= odds ratio) and AIC of models (1, 2 and 4)

	Mode 1			Model 2			Model 4		
Age group	*τ*	*β*	AIC	*τ*	*β*	AIC	*ν*	*β*	AIC
Lowlands									
0-1	8.5	8.25	1362.3	8.20	3.45	1338.1	0.60	1.04	1343.1
2-3	9.2	12.68	1493.3	8.66	4.74	1455.1	0.77	1.01	1461.4
4-5	8.9	12.94	1230.2	8.73	5.65	1191.6	0.84	1.01	1189.6
6-9	8.4	20.29	1522.7	8.13	7.87	1464.1	1	1.01	1477.6
10-19	7.3	9.87	2187.4	6.81	2.90	2138.7	0.64	1.06	2154.5

Highlands									
0-1	7.4	6.49	958.0	7.12	2.16	950.6	0.28	1.40	950.1
2-3	7.5	15.96	1095.9	5.71	2.05	1078.2	0.38	1.30	1080.1
4-5	6.7	19.89	912.8	6.46	3.76	890.5	0.50	1.21	894.4
6-9	5.6	13.33	1322.8	4.63	2.14	1271.6	0.34	1.62	1277.2
10-19	5.3	12.55	1879.8	4.62	2.31	1822.8	0.24	2.20	1847.7

**Figure 3:**
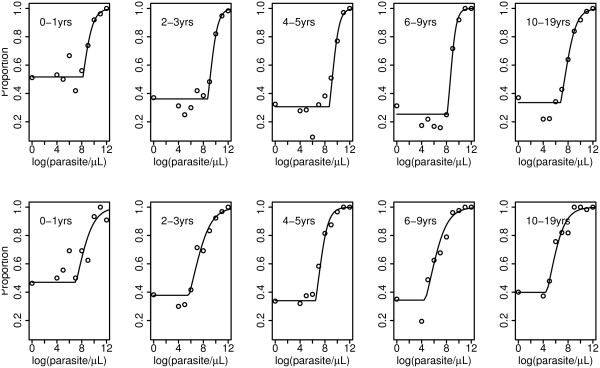
**Observed (open circles) and fitted (lines) proportions of measured fever against log parasite/*μ*L by age group**. Upper and lower rows represent lowlands and highlands, respectively, while the turning point in each line is the threshold value.

So, model (2) was chosen as the best model and hence, results reported hereafter are based on model (2), unless stated. Data were stratified into five age groups, 0-1, 2-3, 4-5, 6-9, and 10-19 years. Figure 3 shows the fitted rate for fever against log(parasite) density, and the threshold parameter is where the curve changes abruptly. The lowest parasite threshold in the lowlands was log(parasite) 6.81 (903 parasite/*μ*L), while in the highlands the lowest was log(parasite) 4.61 (101 parasite/*μ*L).

**Figure 4:**
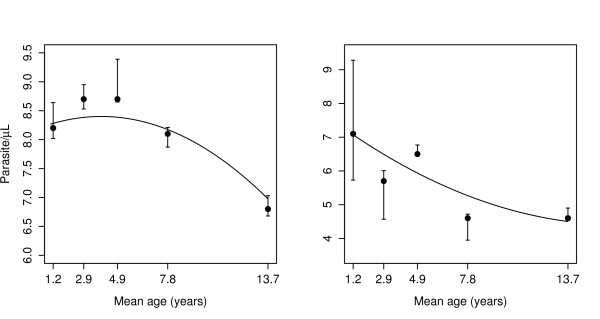
**Distribution of parasite thresholds against mean age groups for lowlands (left) and highlands (right)**. Points represent estimates with 95%CI (line segments) from model (2) and full line is the fit by model (3)

In lowlands, children aged 4-5 years had highest parasite threshold 8.73 (6215 parasite/*μ*L), while the lowest was for those of age group 10-19 years (903 parasite/*μ*L). Children in age group 0-1 in the highlands had the highest parasite threshold 7.12(1233 parasite/*μ*L), while the lowest was seen in oldest age group, see Table 3 and Figure 4. Highland stratum had lower parasite thresholds compared the lowlands, and in both strata thresholds were decreasing with increasing age. Lower 95%CI of parasite threshold in individuals aged 10-19 years in the lowlands villages was 6.68 (796 parasite/*μ*L), while for other age groups in the same stratum, the lower 95%CI was above 7.87(2617 parasite/*μ*L). In the highlands, children aged 0-1 and 4-5 years had lower 95%CI of parasite threshold above 300, while the older age groups, the lower 95%CI threshold were below 100 parasites per *μ*L, see Table 3. Figure 3 shows that in both strata, children in age group 0-1 years who had negative blood smears had higher rate of fever compared to older age groups; and this was decreasing as age increased. A slightly increase in rate was also seen in individuals in age group 10-19 years. This however cannot be interpreted as prevalence of fevers in these age groups within populations, because of sampling fraction between controls and cases which is unknown.

**Table 3:** Estimates of parasite threshold (*τ*) and the sensitivity(%) and specificity(%) with respective 95% Confidence intervals (95%CI).

	Log parasite (parasite/*μ*L) and 95%CI			Sensitivity (95%CI)			Specificity (95%CI)		
	
Age group	*τ*(exp(*τ*))	lower	Upper	**Est**.	lower	Upper	**Est**.	lower	Upper
Lowlands									
0-1 yrs	8.20 (3641)	8.02(3041)	8.64(5653)	74.3	70.7	76.0	67.3	66.3	78.6
2-3 yrs	8.66 (5756)	8.53(5064)	8.95(7708)	74.1	71.4	74.8	76.9	73.1	81.1
4-5 yrs	8.73 (6215)	8.65(5710)	9.39(11968)	64.9	60.1	65.6	86.2	85.9	92.9
6-9 yrs	8.13 (3405)	7.87(2618)	8.21(3678)	69.1	69.1	69.8	90.4	88.3	91.1
10-19 yrs	6.81 (903)	6.68(796)	7.03(1130)	68.3	67.2	69.7	82.8	81.8	86.0

Highlands									
0-1 yrs	7.12 (1233)	5.73(308)	9.28(10721)	67.1	50.6	78.5	66.7	50.0	93.3
2-3 yrs	5.71 (302)	4.57(97)	6.01(407)	91.9	90.4	95.6	53.2	29.8	59.6
4-5 yrs	6.46 (640)	6.44(626)	6.77(871)	84.0	84.0	84.0	72.7	71.2	80.3
6-9 yrs	4.63 (102)	3.95(52)	4.72(112)	96.9	96.9	99.1	37.2	16.7	37.2
10-19 yrs	4.62 (101)	4.54(94)	4.90(134)	91.9	90.3	91.9	47.2	47.2	61.8

Parameters were estimates by profile likelihood methods using model (2)

**Figure 5:**
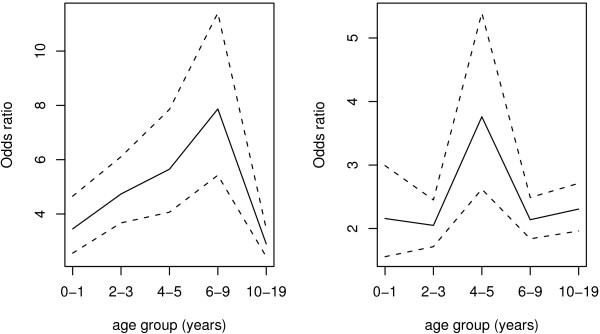
**Distribution of odds ratios (full lines) and 95%CI (dashed lines) for fever by age group among individuals with parasite exceeding threshold (*τ*) shown in Table (2)**. The left and right plots are for lowland and highland strata, respectively.

Figure 5 shows that odds ratio (OR) of fever in individuals who had parasite above the threshold value was increasing across the age group in the lowlands, where the maximum was reached in the age group 6-9 years. There were more or less similar ORs in the highlands, except in age group 4-5 years, which had higher OR than rest of age groups.

Results from model (3) showed that, parasite threshold varied significantly with age in both strata. In the lowlands, the threshold parameter was log parasites density 8.164(95%CI 7.990 - 9.071), and it was increasing by 0.119(95%CI: -0.092 - 0.174) when mean age increased by one year, and decreased by 0.015(95%CI: 0.003 - 0.019) for any increase in mean age squared by one year squared, see Figure 4. Similarly, in the highlands the threshold parameter was log parasite density 7.480(95%CI: 6.942 -8.326), which decreased by 0.368(95%CI: 0.221-0.604) when mean age increased by one year and increased by 0.011(95%CI: 0.002 - 0.024) with the increase of mean age squared by one year squared. In the lowlands stratum, the highest and lowest parasite threshold were (4397 parasites/*μ*L) and (1071 parasites/*μ*L) in age group 4-5 and 10-19 years, respectively. This model shows that there was a significant age effect in both strata and that the threshold parameters estimated from this model were similar to the ones obtained by model (2).

Sensitivity and specificity of threshold parameter for different age groups and strata is as shown in Table 3. The sensitivity were higher in the highlands (range 67%-97%) than in the lowlands (range 65%-74%), while specificity were higher in the lowlands (range 67%-90%) than in the highlands (range 37%-73%). In the lowlands, children aged 0-1 years had the highest sensitivity and lowest specificity, while in the highlands, the highest sensitivity and lowest specificity, were in children of age 6-9 years. On average, in the lowlands the specificity was higher than sensitivity while in the highlands it was the opposite.

## Discussion

A better understanding of interaction between malaria parasites and clinical features might lead to a better designing and evaluation of different malaria interventions particularly drugs and vaccines. The main aim of our analysis was to determine the parasite threshold associated with fever in individuals aged 0-19 years. Lowland stratum is characterized as high malaria transmission area and the highlands as the low transmission area [16,26], and that in the high transmission area, children are the most affected while in the low transmission area all age groups are almost equally affected because of lack of acquired immunity against parasite and clinical disease [5]. The acquired malaria immunity varies with the level of exposure to parasite; hence individuals living in holoendemic areas tend to acquire immunity sooner than those in hyper endemic areas. Older individuals in holoendemic areas have higher immunity than children because of their prolonged exposure to malaria parasites [9].

Our modeling approach used strata and age of individuals as an important factors in determining parasite threshold associated with fever. We fitted three models (1-3) to determine parasite thresholds. Model (3) is an extension of model (2), where instead of fitting different models for each age group, one model was fitted for the whole data taking age as explanatory variable. A fourth model (4) was fitted for the purpose of assessing whether the first two models fits the data well when compared to the traditional ones. Model (1 and 2) had low information compared to model (4) because of the truncation (i.e parasite below and/or above *τ *were each categorized in one group), and hence the first model had lowest information.

Comparing the AICs, model (1) performed poorly when compared to the other two models, and this indicates that grouping parasite density into two groups is an oversimplification and might results in poor predictions. It was interesting to note that model (2) performs better than model (4) for most of groups (the exception being lowlands 4-5 and highlands 0-1 years) despite the fact that model (2) was based on a cut-off threshold and therefore truncated data. This probably reflects that quantification of low density parasitaemia by microscopy is difficult and not precise.

Simulation shows that model (2) was better in the estimation of malaria parasite threshold than model (1). The model provides the background level where the probability of fever is considered similar for parasite below the threshold and then probability of fever increases as parasite density increases above the threshold. Model (1) might be simple but this gives only the probabilities below threshold and that above the threshold, and it does not give a flexible way of choosing the threshold; for instance when a strict parasite threshold is desired. Furthermore, the model is weak especially when the slope parameter (rate of change of parasite density) is low, as it can overestimate the threshold parameter, see Table 1.

Model (3) gives a better way of explaining the relation of the thresholds to variable of interest. For example, the age and transmission intensities which are known to be important determinants in malaria transmission. This model provides a way of adjusting for other covariates. However, the problem is the optimization process where computing time grows as number of parameters to be estimated increases. For example, in a computer with Intel Pentium^® ^Dual CPU T3200 2.0 GHz processor and 4 GB of RAM, without sub-sampling bootstrap, it takes few seconds to get the estimates for the parameters in models (1 and 2), while model in(3) where *τ *was fitted on age and age squared, in a data set with 5050 records it requires about 5 minutes. Threshold parameters were well estimated in model (3) especially for the lowlands strata, as can be seen from Figure 4; that a uniform confidence band will also include the fitted estimates of thresholds in the separate age groups. However, in the highlands, there was much variability in the threshold parameters fitted by model (2), which might be due to the few number of individuals with positive smears in each age group. So, model (3) might be more appropriate since it contains more data. Source of variations in the model fittings can be due to measurement errors in parasite density, which could be due to inaccurate parasite quantification as a result of either poor sample preparation, error in parasite counting and technician performance [27].

Even though methods used in estimation of parasite threshold and our data set type differ considerably with other studies [10,28], the findings from this study are still comparable. For example, in children below 5 years, Chandler et., al. [28] found a parasite threshold of 4000 parasite/*μ*L in those living in the lowlands and 1000 in the the highlands, while Smith et., al. [10] found a threshold of 5000 in children living in area of high transmission. The parasite thresholds were higher in high transmission area and lower in low transmission area [19,28]. There was also a difference in the threshold across the age groups where children below five years had higher parasite threshold compared to older individuals, which is similar pattern as found elsewhere [28]. This study shows that children in age group 2-5 years in the lowlands had the highest threshold while individuals in age group 10-19 years had the lowest. The pattern of the thresholds in lowlands, where malaria is endemic shows a similar trend to that of parasite density by age. This suggests that immunity plays a significant role in the threshold, as it has been shown to develop slowly in individuals who are constantly exposed to malaria, where a period of ten years was estimated to be the maximum for full development of immunity [29].

Results from sensitivity and specificity analysis for case definition using parasite threshold gave results similar to other studies done in lowland areas [10,28], suggesting that the model can be used in similar settings.

## Conclusion

We conclude that dose response model with threshold parameter can be used to estimate parasite thresholds. Parasite threshold varies with transmission intensity, and in this area children below five years have highest threshold.

## Competing interests

The authors declare that they have no competing interests.

## Authors' contributions

BPM, TGT and THS developed conception and design of the study. JPL, LSV, MML and TGT designed malariometric study and commented on the manuscript. BPM and THS contributed with the statistical analysis and interpretation of results. BPM wrote the manuscript. All authors read and approved the final version for publication.

## Pre-publication history

The pre-publication history for this paper can be accessed here:

http://www.biomedcentral.com/1471-2288/9/75/prepub

## References

[B1] Ministry of Health Plan of action for implementing Roll Back Malaria in Tanzania 2001 Dar es salaam, Tanzania

[B2] Bojang K Van Hensbroek M Palmer A Banya W Jaffar S Greenwood B Predictors of mortality in Gambian children with severe malaria anaemia Annal of Tropical Paediatrics 1997 17 4 355 9 10.1080/02724936.1997.11747910 9578796

[B3] Kahigwa E Schellenberg D Sanz S Aponte J Wigayi J Mshinda H Alonso P Menendez C Risk factors for presentation to hospital with severe anaemia in Tanzanian children: a case-control study Tropical Medicine & International Health 2002 7 10 823 830 10.1046/j.1365-3156.2002.00938.x 12358616

[B4] Marsh K Malaria disaster in Africa The Lancet 1998 352 9132 10.1016/S0140-6736(05)61510-3 9752813

[B5] Greenwood B Fidock D Kyle D Kappe S Alonso P Collins F Duffy P Malaria: progress, perils, and prospects for eradication The Journal of Clinical Investigation 2008 118 4 1266 1276 10.1172/JCI33996 18382739 PMC2276780

[B6] Kirby M Milligan P Conway D Lindsay S Study protocol for a three-armed randomized controlled trial to assess whether house screening can reduce exposure to malaria vectors and reduce malaria transmission in The Gambia Trials 2008 9 33 10.1186/1745-6215-9-33 18538004 PMC2427015

[B7] Hatcher J Delea K Richardson J Pennell K Miller G Disruption of dopamine transport by DDT and its metabolites Neurotoxicology 2008 29 4 682 690 10.1016/j.neuro.2008.04.010 18533268 PMC4755343

[B8] Todryk S Hill A Malaria vaccines: the stage we are at Nature Reviews Microbiology 2007 5 7 487 489 10.1038/nrmicro1712 17571459

[B9] Lusingu J Vestergaard L Mmbando B Drakeley C Jones C Akida J Savaeli Z Kitua A Lemnge M Theander T Malaria morbidity and immunity among residents of villages with different Plasmodium falciparum transmission intensity in North-Eastern Tanzania Malaria Journal 2004 3 26 15282030 10.1186/1475-2875-3-26 PMC514496

[B10] Smith T Armstrong Schellenberg J Hayes R Attributable fraction estimates and case definitions for malaria in endemic areas Statistics in Medicine 1994 13 22 2345 2358 10.1002/sim.4780132206 7855468

[B11] Koram K Molyneux M When Is" Malaria" Malaria? The Different Burdens of Malaria Infection, Malaria Disease, and Malaria-Like Illnesses The American Journal of Tropical Medicine and Hygiene 2007 77 6 Suppl 1 5 18165468

[B12] Rogier C Commenges D Trape J Evidence for an Age-Dependent Pyrogenic Threshold of Plasmodium falciparum Parasitemia in Highly Endemic Populations The American Journal of Tropical Medicine and Hygiene 1996 54 6 613 619 8686780 10.4269/ajtmh.1996.54.613

[B13] Yanagimoto T Yamamoto E Estimation of safe doses: critical review of the hockey stick regression method Environ Health Perspect 1979 32 193 199 10.2307/3429016 540593 PMC1637920

[B14] Cox C Threshold dose-response models in toxicology Biometrics 1987 43 3 511 23 10.2307/2531991 3663815

[B15] Schwartz P Gennings C Teuschler L Fariss M Optimizing the Precision of Toxicity Threshold Estimation Using a Two-Stage Experimental Design Journal of Agricultural, Biological & Environmental Statistics 2001 6 4 409 428 10.1198/10857110152946802

[B16] Bødker R Akida J Shayo D Kisinza W Msangeni H Pedersen E Lindsay S Relationship Between Altitude and Intensity of Malaria Transmission in the Usambara Mountains, Tanzania Journal of Medical Entomology 2003 40 5 706 717 10.1603/0022-2585-40.5.706 14596287

[B17] Lusingu J Jensen A Vestergaard L Minja D Dalgaard M Gesase S Mmbando B Kitua A Lemnge M Cavanagh D Hviid L Theander T Levels of plasma immunoglobulin G with specificity against the cysteine-rich interdomain regions of a semiconserved Plasmodium falciparum erythrocyte membrane protein 1, VAR4, predict protection against malarial anemia and febrile episodes Infection and immunity 2006 74 5 2867 10.1128/IAI.74.5.2867-2875.2006 16622225 PMC1459698

[B18] Mmbando B Segeja M Msangeni H Sembuche S Ishengoma D Seth M Francis F Rutta A Kamugisha M Lemnge M Epidemiology of malaria in an area prepared for clinical trials in Korogwe, north-eastern Tanzania Malaria Journal 2009 8 165 10.1186/1475-2875-8-165 19615093 PMC2720983

[B19] Mwangi T Ross A Snow R Marsh K Case definitions of clinical malaria under different transmission conditions in Kilifi District, Kenya Journal of Infectious Diseases 2005 191 11 1932 1939 10.1086/430006 15871128 PMC3545188

[B20] McGuinness D Koram K Bennett S Wagner G Nkrumah F Riley E Clinical case definitions for malaria: clinical malaria associated with very low parasite densities in African infants Transactions of the Royal Society of Tropical Medicine and Hygiene 1998 92 5 527 531 10.1016/S0035-9203(98)90902-6 9861370

[B21] Box G Tidwell P Transformation of the independent variables Technometrics 1962 4 4 531 550 10.2307/1266288

[B22] Politis D Romano J Wolf M Subsampling for heteroskedastic time series Journal of Econometrics 1997 81 2 281 317 10.1016/S0304-4076(97)86569-4

[B23] Csorgo M Horvath L Limit theorem in change point analysis 1997 New York: Wiley

[B24] Wilson K Bjørnstad O Dobson A Merler S Poglayen G Randolph S Read A Skorping A Hudson P, Rizzoli B, Grenfell B, Heesterbeek H, Dobson A Heterogeneities in macroparasite infections: patterns and processes The Ecology of Wildlife Diseases 2002 New York: Oxford University Press 6 44

[B25] Pepe S The Statistical Evaluation of Medical Tests for Classification and Prediction 2003 New York: Oxford University Press

[B26] Drakeley C Carneiro I Reyburn H Malima R Lusingu J Cox J Theander T Nkya W Lemnge M Riley E Altitude-dependent and-independent variations in Plasmodium falciparum prevalence in Northeastern Tanzania Journal of Infectious Diseases 2005 191 10 1589 1598 10.1086/429669 15838785

[B27] O'meara WP Mckenzie FE Magill AJ Forney JR Permpanich B Lucas C Gasser J Robert A Wongsrichanalai C Sources of variability in determining malaria parasite density by microscopy American Journal of Tropical Medicine & Hygiene 2005 73 3 593 598 PMC2500224 16172488

[B28] Chandler C Drakeley C Reyburn H Carneiro I The effect of altitude on parasite density case definitions for malaria in northeastern Tanzania Tropical Medicine & International Health 2006 11 8 1178 1184 10.1111/j.1365-3156.2006.01672.x 16903881

[B29] Klein E Smith D Boni M Laxminarayan R Clinically immune hosts as a refuge for drug-sensitive malaria parasites Malaria Journal 2008 7 67 10.1186/1475-2875-7-67 18439283 PMC2409364

